# Salvianolic Acid B Alleviates MAFLD by Targeting PPAR‐α: Mechanistic Insights From Network Pharmacology and Lipidomics

**DOI:** 10.1002/fsn3.71646

**Published:** 2026-03-19

**Authors:** Fengyan Huang, Chen Qiu, Danna Wang, Yuanying Ni, Zhuotao Fu, Linchun Fu, Chao Liang, Shangyi Huang, Zhitong Deng

**Affiliations:** ^1^ The Second Clinical College of Guangzhou University of Chinese Medicine Guangzhou China; ^2^ The First Affiliated Hospital of Guangzhou University of Chinese Medicine Guangzhou China; ^3^ The First Affiliated Hospital of Jinan University Guangzhou China; ^4^ The Fifth College of Clinical Medicine Guangzhou University of Chinese Medicine Guangzhou China; ^5^ Science and Technology Innovation Center Guangzhou University of Chinese Medicine Guangzhou China; ^6^ Haikou Hospital of Traditional Chinese Medicine Haikou China

**Keywords:** fatty acid oxidation, food‐derived bioactive compound, inflammation, lipid metabolism, MAFLD, oxidative stress, PPAR‐α/PGC‐1α, salvianolic acid B

## Abstract

Metabolic dysfunction‐associated fatty liver disease (MAFLD) remains a global health burden with limited definitive therapies, highlighting the need for safe, food‐derived interventions. Salvianolic acid B (SALB), a major water‐soluble bioactive component of the traditional Asian health‐promoting food *Salvia miltiorrhiza Bunge*, exhibits lipid‐lowering, anti‐inflammatory, and antioxidant properties, but its therapeutic potential and mechanisms in MAFLD remain unclear. Here, we employed an integrated approach combining network pharmacology, molecular docking, surface plasmon resonance affinity assays, lipidomics, and experiments in vitro and in vivo to address this gap. Network pharmacology combined with lipidomics identified PPAR‐α as a key target of SALB. Molecular docking and SPR assays confirmed direct binding between SALB and PPAR‐α. In vitro, SALB reduced triglyceride levels and lipid accumulation in HepG2 cells, enhanced fatty acid oxidation (FAO), and upregulated PPAR‐α, PGC‐1α, and FAO‐related genes (CPT1, CPT2, ACADL, ACADVL). In HFD‐fed mice, SALB decreased serum total cholesterol, triglycerides, LDL‐C, ALT, AST, while increasing HDL‐C. Additionally, SALB upregulated hepatic PPAR‐α and FAO‐related gene expression and suppressed hepatic reactive oxygen species production and inflammatory responses in both models. Collectively, our findings demonstrate that SALB, a natural food‐derived bioactive compound, targets PPAR‐α to ameliorate MAFLD by enhancing FAO, modulating lipid metabolism, and mitigating oxidative stress and inflammation. This work supports SALB's potential as a dietary supplement for MAFLD and metabolic disease management, reinforcing the value of exploring functional components from health‐promoting food.

## Graphical abstract

Salvianolic acid B (SALB) is the major water‐soluble bioactive constituent of *Salvia miltiorrhiza Bunge*, a traditional Asian health‐promoting food, while its therapeutic potential and molecular mechanisms in the pathogenesis of MAFLD remain largely unclear. To fill this research gap, we adopted an integrated strategy encompassing network pharmacology, lipidomics, molecular docking, surface plasmon resonance affinity assays, as well as in vitro and in vivo experimental validations to systematically investigate the role of SALB in MAFLD. Collectively, our findings revealed that SALB, a natural food‐derived, bioactive compound, exerts a protective effect against MAFLD by targeting PPAR‐α, which in turn enhances fatty acid oxidation, modula tes lipid metabolic homeostasis, and alleviates oxidative stress and inflammatory responses. These results indicate that SALB holds great promise as a dietary supplement for the prevention and management of MAFLD and other metabolic disorders.

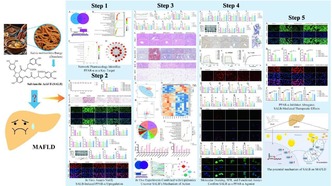

**Abbreviations**
ACADL: acyl‐CoA dehydrogenase, long chain
ACADVL: acyl‐CoA dehydrogenase very long chain
ALT: Alanine aminotransferase
AST: aspartate aminotransferase
BCA: bicinchoninic acid
BP: biological process
CC: cellular component
CCK‐8: cell counting Kit‐8
cDNA: complementary DNA
CPT1: carnitine palmitoyltransferase 1
CPT2: carnitine palmitoyltransferase 2
DL: drug‐likeness
DMEM: Dulbecco's modified Eagle's medium
ECL: enhanced chemiluminescence
ECM1: Extracellular Matrix Protein 1
FAO: fatty acid oxidation
FDA: Food and Drug Administration
GO: gene ontology
HDL‐C: high‐density lipoprotein cholesterol
H&E: hematoxylin and eosin
HFD: high‐fat diet
HRP: horseradish peroxidase
IHC: immunohistochemical
KEGG: Kyoto Encyclopedia of Genes and Genomes
LC/MS: ultra‐performance liquid chromatography‐mass spectrometry
LDL‐C: low‐density lipoprotein cholesterol
MAFLD: metabolism‐associated fatty liver disease
MF: molecular function
NAFLD: non‐alcoholic fatty liver disease
OA: oleic acid
OCA: obeticholic acid
PA: palmitic acid
PBS: phosphate‐buffered saline
PFA: paraformaldehyde
PGC‐1α: PPARγ coactivator‐1α
PPAR‐α: peroxisome proliferator‐activated receptor alpha
PPI: protein–protein interaction
PVDF: polyvinylidene difluoride
qRT‐PCR: quantitative real‐time polymerase chain reaction
SALB: Salvianolic acid B
TC: total cholesterol
TCMSP: Traditional Chinese Medicine Systems Pharmacology
TG: triglyceride
TGF‐β: transforming growth factor‐beta
VLDLs: very low‐density lipoproteins

## 1 Introduction

Metabolism‐associated fatty liver disease (MAFLD) has become a pressing global health challenge, affecting approximately one‐third of the world's population (Lou et al. 2024). Characterized by hepatic steatosis, hepatocellular injury, and chronic inflammation, MAFLD can progress to fibrosis, cirrhosis, and even hepatocellular carcinoma if left untreated (Duell et al. 2022; Eslam et al. 2020; Rinella et al. 2023; Sun et al. 2023; Yilmaz et al. 2021). At its core, MAFLD pathogenesis is driven by dysregulated lipid metabolism, a complex process governed by fatty acid uptake and output, neo‐lipogenesis, and β‐fatty acid oxidation on fat utilization (Badmus et al. 2022). Thus, therapeutic strategies targeting hepatic lipid accumulation have emerged as crucial interventions for MAFLD management.

Peroxisome proliferator‐activated receptor alpha (PPAR‐α) is a member of the ligand‐activated nuclear hormone receptor superfamily (Gorla‐Bajszczak et al. 1999), predominantly expressed in the liver. This nuclear receptor is critical for lipid homeostasis, regulating β‐oxidation, fatty acid transport, and energy metabolism via fatty acid oxidation (FAO) (Hinds et al. 2017; Scarpulla 2008; Schreurs et al. 2010). Its transcriptional activation requires the binding of specific ligands (Pawlak et al. 2015; Tahri‐Joutey et al. 2021), and recruitment of transcriptional coactivators such as peroxisome proliferator‐activated receptor gamma coactivator 1‐alpha (PGC‐1α), a master regulator of mitochondrial biogenesis and function (Haemmerle et al. 2011; Vega et al. 2000). Mitochondrial FAO process is heavily reliant on carnitine palmitoyltransferase 1 (CPT1), which enables mitochondrial fatty acid transport—the rate‐limiting step in long‐chain FAO (Miguel et al. 2021; Schlaepfer and Joshi 2020; Su et al. 2023). Additionally, in mitochondrial β‐oxidation, acyl‐CoA dehydrogenase (ACADL) initiates the first enzymatic reaction (Softic et al. 2019). PPAR‐α activation also influences systemic lipid metabolism by reducing circulating triglyceride concentrations while elevating high‐density lipoprotein (HDL) levels, thereby promoting ketogenesis (Hughes et al. 2006; van Raalte et al. 2004). Beyond metabolic regulation, PPAR‐α activation exerts anti‐inflammatory and antioxidant effects in the liver and other tissues, breaking the vicious cycle between lipid dysregulation and inflammation that exacerbates MAFLD (Reda et al. 2023; Shen et al. 2020). Currently, the only FDA‐approved drug for this indication is Resmetirom (Khare et al. 2025), a novel thyroid hormone receptor‐β selective agonist. However, its clinical utility is limited by suboptimal response rates and safety concerns (Caddeo and Romeo 2025), highlighting the need for novel, well‐tolerated therapeutic agents targeting PPAR‐α‐mediated pathways.

*Salvia miltiorrhiza Bunge*, a traditional Asian health‐promoting food (Li, Meng, et al. 2025; Raposo et al. 2021), is rich in salvianolic acid B (SALB), a major water‐soluble bioactive component (Xu et al. 2022). Previous studies have demonstrated SALB's protective effects against hepatic injury, ferroptosis, and lipid metabolism disorders, attributed to its antioxidant (Wang et al. 2015), anti‐inflammatory (Meng et al. 2022), and gut microbiota‐regulating properties (Wang et al. 2024). However, the specific pathways through which SALB regulates lipid metabolism remain poorly characterized, and direct evidence linking SALB to PPAR‐α activation is lacking.

To address this critical gap, we employed an integrated approach combining network pharmacology, lipidomics, molecular docking, surface plasmon resonance (SPR) affinity assays, and PPAR‐α inhibitor (GW6471) validation. Using high‐fat diet (HFD)‐fed C57BL/6 mice and oleic/palmitic acid‐treated HepG2 cells, we systematically investigated SALB's therapeutic efficacy and molecular mechanism in MAFLD. Our study aimed to test the hypothesis that SALB targets PPAR‐α to enhance FAO, modulate lipid metabolism, and mitigate oxidative stress and inflammation. This work provides novel mechanistic insights into SALB—a natural food‐derived bioactive compound—positioning it as a promising candidate for MAFLD intervention and reinforcing the value of exploring functional components from edible‐medicinal herbs for metabolic disease management.

## 2 Materials and Methods

### 2.1 Network Pharmacology Analysis

The potential therapeutic targets of SALB were systematically identified through comprehensive searches in multiple public databases, including TCMSP (http://tcmspw.com/), HERB (http://herb.ac.cn/) and GeneCards (https://www.genecards.org/). Simultaneously, the search terms “MAFLD” and “NAFLD (non‐alcoholic fatty liver disease)” were used to obtain the targets linked to MAFLD. Using the UniProt database (http://www.uniprot.org/), all of the identified targets were then standardized to their official human gene nomenclature. The intersection between SALB‐related targets and MAFLD‐associated targets was determined and graphically represented using a Venn diagram. To elucidate the biological significance of these overlapping targets, we performed Gene Ontology (GO) functional enrichment analysis and Kyoto Encyclopedia of Genes and Genomes (KEGG) pathway analysis using the Metascape database (http://metascape.org/). To highlight the most significantly enriched terms, the enrichment results were visualized as bubble charts. In addition, protein–protein interaction (PPI) networks were generated using the STRING database and analyzed with Cytoscape software (version 3.10.1).

### 2.2 Chemicals and Reagents

SALB (GB4051) was purchased from Tsingke Biotechnology Co. Ltd. (Beijing, China). High Fat Diet (HFD) feed (60%; GD60) was purchased from Guangdong Medical Laboratory Animal Center (Guangdong, China). A fatty acid oxidation enzyme‐linked immunosorbent assay kit (ab118182) was purchased from Abcam. Oleic acid (OA; 75,090), palmitic acid (PA; P5585), and Oil Red O staining solution (O0625‐25 g) were purchased from Sigma‐Aldrich. Hematoxylin and eosin (H&E) dye solutions were obtained from Regen Biotech (Beijing, China). Biochemical assay kits for alanine aminotransferase (ALT; C009‐2‐1), aspartate aminotransferase (AST; C010‐2‐1), triglyceride (TG; A110‐1‐1), total cholesterol (TC; A111‐1‐1), low‐density lipoprotein cholesterol (LDL‐C; A113‐1‐1), and high‐density lipoprotein cholesterol (HDL‐C; A112‐1‐1) assay kits were provided by Jiancheng Institute of Biotechnology (Jiangsu, China). The BODIPY 493/503 Staining Kit (C2053S) was acquired from Beyotime Biotechnology (Beijing, China). All enzyme‐linked immunosorbent assay (ELISA) kits were purchased from Ruixin Biotechnology Company (Guangzhou, China). DCFH‐DA Reactive Oxygen Fluorescent Probe (Red, M176010) was purchased from MREDA Technology Co. Ltd. (Beijing, China). GW6471 (C16599031) was purchased from Macklin Biochemical Technology Co. Ltd. (Shanghai, China). TRIzol reagent and RNA Storage Solution were provided by Invitrogen (10,296,028, AM7000, USA). ABScript III RT Master Mix for qPCR with gDNA Remover (RK20433) and Universal SYBR Green Fast qPCR Mix (RK21203) were provided by ABclonal (Hubei, China). RIPA Lysis Buffer and a bicinchoninic acid (BCA) protein quantification kit were obtained from Beyotime Biotechnology. Primary antibodies against PPAR‐α (A18252) and PGC‐1a (A11971) were obtained from ABclonal, while CPT1A (15184‐1‐AP) and β‐actin (81115‐1‐RR) were purchased from Proteintech (Hubei, China), and NF‐κB p65 (AB2020) was purchased from Beyotime Biotechnology (Shanghai, China). Anti‐rabbit (CSB‐PA564648) and anti‐mouse (CSB‐PA644737) horseradish peroxidases were purchased from CUSABIO (Hubei, China). Pre‐stained Protein Marker (8–200 kDa; G2083) was obtained from Service Bio (Hubei, China).

### 2.3 Cell Culture and CCK8 Assay

HepG2 cells were maintained in Dulbecco's modified Eagle's medium (DMEM) containing 10% fetal bovine serum under standard culture conditions (37°C, 5% CO_2_). After 24 h of seeding, cells were exposed to 50 μM PA and 200 μM OA for an additional 24 h. The Cell Counting Kit‐8 (CCK‐8) was used to measure the vitality of the cells in accordance with manufacturer instructions (DMEM to CCK‐8 solution ratio: 10:1).

### 2.4 Oil Red O Staining and BODIPY Analysis

HepG2 cells were rinsed twice with phosphate‐buffered saline (PBS) and fixed using 4% paraformaldehyde (PFA; BioSharp, Hefei, China). For Oil Red O staining, cells were incubated with filtered Oil Red O solution for 30 min, followed by washing with 60% isopropanol to remove excess stain and three rinses with PBS. In the case of BODIPY analysis, cells were washed twice with PBS, and the procedure was carried out following the manufacturer's protocol. Digital images were acquired using a microscope or digital pathology slide scanner (KF‐PRO‐005‐EX; KFBIO, Zhejiang, China).

### 2.5 Reactive Oxygen Species (ROS) Analysis

ROS detection was performed following established procedures (Sun et al. 2021). For tissue analysis, fresh liver samples were processed within 2 h post‐excision to generate 10‐μm‐thick unfixed cryosections. After being kept at −20°C in the presence of mild protection, these sections were stained again. Tissue sections were incubated with a Cellular Reactive Oxygen Species Detection Assay Kit (red fluorescence) at 37°C in a dark environment for 30 min, followed by PBS rinses, mounted with glycerol, and fluorescence microscopy visualization. HepG2 cells were grown on sterile glass coverslips in 24‐well plates and given the option to attach overnight in order to quantify cellular ROS. After treatment with SALB at indicated concentrations, cells were fixed with 4% PFA for 15 min, loaded with DCFH‐DA probe at 37°C for 30 min in the dark. Following three washes with PBS, cells were incubated with DAPI.

### 2.6 Immunofluorescence Staining

HepG2 cells were firstly fixed with PFA, followed by permeabilization with Triton X‐100, blocked with 2% bovine serum albumin, and incubated with primary antibodies against PPAR‐α (1:100) or NF‐κB p65 (1:100) at 4°C overnight. The cells were then incubated with secondary antibodies and stained with DAPI. Images were acquired using a digital pathology slide scanner.

### 2.7 Animals and Treatments

Forty male C57BL/6 J mice aged 4–6 weeks (20–22 g) were purchased from the Animal Experimental Center of the Guangzhou University of Chinese Medicine (Guangdong, China). The Animal experiments were approved by the Animal Ethics Committee of Guangzhou University of Chinese Medicine (Ethics Certificate number, 20250120005). All mice were given free access to food and water and maintained in a 12/12 h light–dark cycle at 25°C. After a week of acclimatization, the mice were randomized into five groups of eight mice each: normal (control), HFD model (HFD), HFD + obeticholic acid (OCA; 30 mg/kg/d), HFD + SALB‐L (10 mg/kg/d), and HFD + SALB‐H (20 mg/kg/d; Meng et al. 2022). The control group was fed a regular chow diet for 12 weeks, whereas the other groups were fed an HFD. During the last 2 weeks, the HFD + OCA, HFD + SALB‐L, and HFD + SALB‐H groups were administered OCA or SALB via gavage at different doses, whereas the control and model groups were administered saline via gavage at the same volume. Serum and tissue samples were collected from the mice on the last day under anesthesia.

### 2.8 Biochemical Analysis

Blood samples were obtained via retro‐orbital bleeding from the mice and subsequently centrifuged at 4000 rpm for 10 min to separate the serum. Concentrations of blood lipids and liver function related indicators in the serum were quantified following the procedures given by the respective assay kits. Serum interleukin (IL)‐1β, IL‐6, and tumor necrosis factor (TNF)‐α were tested using ELISA kits following the manufacturer's instructions. Glutathione (GSH), superoxide dismutase (SOD), and malondialdehyde (MDA) were analyzed using standardized assay procedures as specified in the respective kit instructions.

### 2.9 Histopathological and Immunohistochemical (IHC) Analyses

Hepatic and fat tissues were fixed with 4% PFA, embedded in paraffin, sectioned, and stained with H&E. Some hepatic tissues were frozen at the optimal cutting temperature, sectioned, and stained with Oil Red O to assess the hepatic lipid content. For IHC analysis, the sections were incubated with PPAR‐α (1:200) primary antibody after blocking with 10% bovine serum albumin. After three washes with PBS, the sections were incubated with horseradish peroxidase and developed using diaminobenzidine hydrochloride. Images were acquired using a digital pathology slide scanner.

### 2.10 Real‐Time Quantitative PCR

Total RNA was extracted from specific cell cultures or hepatic tissue specimens using TRIzol reagent, according to the manufacturer's protocol. Subsequently, the RNA was reverse‐transcribed into complementary DNA (cDNA) using a high‐capacity cDNA reverse transcription kit. Quantitative real‐time polymerase chain reaction (qRT‐PCR) analysis was performed using 2× Universal SYBR Green Fast qPCR Mix (RK21203; ABclonal), with β‐actin serving as an endogenous control for gene expression normalization. All primers used are listed in Table 1.

**TABLE 1:** Primers used for the RT‐PCR analysis.

Gene	Sequence 5′‐3′ (forward)	Sequence 5′‐3′ (reverse)
(Mouse) Ppar‐α	AGAGCCCCATCTGTCCTCTC	ACTGGTAGTCTGCAAAACCAAA
(Mouse) Pgc‐1α	TATGGAGTGACATAGAGTGTGCT	CCACTTCAATCCACCCAGAAAG
(Mouse) Cpt1	CTCCGCCTGAGCCATGAAG	CACCAGTGATGATGCCATTCT
(Mouse) Cpt2	CAGCACAGCATCGTACCCA	TCCCAATGCCGTTCTCAAAAT
(Mouse) Acadvl	CTACTGTGCTTCAGGGACAAC	CAAAGGACTTCGATTCTGCCC
(Mouse) Acadl	TCTTTTCCTCGGAGCATGACA	GACCTCTCTACTCACTTCTCCAG
(Mouse) NF‐κB	GGAGGCATGTTCGGTAGTGG	CCCTGCGTTGGATTTCGTG
(Mouse) IL‐1β	GCAACTGTTCCTGAACTCAACT	ATCTTTTGGGGTCCGTCAACT
(Mouse) IL‐6	TAGTCCTTCCTACCCCAATTTCC	TTGGTCCTTAGCCACTCCTTC
(Mouse) TNF‐α	CCTCTCTCTAATCAGCCCTCTG	GAGGACCTGGGAGTAGATGAG
(Mouse) Cox‐2	TTCAACACACTCTATCACTGGC	AGAAGCGTTTGCGGTACTCAT
(Mouse) β‐Actin	ATGACCCAAGCCGAGAAGG	CGGCCAAGTCTTAGAGTTGTTG
(Human) PPAR‐α	ATGGTGGACACGGAAAGCC	CGATGGATTGCGAAATCTCTTGG
(Human) PGC‐1α	TCTGAGTCTGTATGGAGTGACAT	CCAAGTCGTTCACATCTAGTTCA
(Human) CPT1	TCCAGTTGGCTTATCGTGGTG	TCCAGAGTCCGATTGATTTTTGC
(Human) CPT2	CATACAAGCTACATTTCGGGACC	AGCCCGGAGTGTCTTCAGAA
(Human) ACADVL	ACAGATCAGGTGTTCCCATACC	CTTGGCGGGATCGTTCACTT
(Human) ACADL	AGGGGATCTGTACTCCGCAG	CTCTGTCATTGCTATTGCACCA
(Human) NF‐κB	GGAGGCATGTTCGGTAGTGG	CCCTGCGTTGGATTTCGTG
(Human) IL‐1β	GCAACTGTTCCTGAACTCAACT	ATCTTTTGGGGTCCGTCAACT
(Human) IL‐6	TAGTCCTTCCTACCCCAATTTCC	TTGGTCCTTAGCCACTCCTTC
(Human) TNF‐α	CCTCTCTCTAATCAGCCCTCTG	GAGGACCTGGGAGTAGATGAG
(Human) Cox‐2	TTCAACACACTCTATCACTGGC	AGAAGCGTTTGCGGTACTCAT
(Human) β‐Actin	ACAGAGCCTCGCCTTTGC	GATATCATCATCCATGGTGAGCTGG

### 2.11 Western Blotting Analysis

Protein samples were prepared from the indicated cell cultures or liver tissues using RIPA lysis buffer, followed by homogenization and quantification using a BCA protein assay. Equal amounts of protein (80–100 μg) were resolved using 10% sodium dodecyl sulfate‐polyacrylamide gel electrophoresis and subsequently transferred onto polyvinylidene fluoride membranes. For immunoblotting, the membranes were probed with primary antibodies against PPAR‐α, PGC‐1α, CPT1A, and β‐actin. Antibody incubation was performed according to the manufacturer's instructions.

### 2.12 Lipid Metabolomics Analysis

Six mice from each of the HFD and HFD + SLAB‐H groups were selected for lipid metabolomic analysis using ultra‐performance liquid chromatography‐mass spectrometry (LC/MS) (Shanghai LuMing Biological Technology Co. Ltd., Shanghai, China). Briefly, the samples were separated using a Nexera UPLC (Shimadzu, Kyoto, Japan) and analyzed using mass spectrometry with a Q Exactive (Thermo Fisher Scientific). Heated electrospray ionization was used for detection in positive and negative ion modes. LC/MS data were processed using Progenesis QI software (version 2.3, Nonlinear Dynamics, Newcastle, UK) for baseline filtering, peak detection, integration, retention time correction, peak alignment, and normalization. Lipid classes involved in this study included PC, TG, PEt, PE, SM, PG, PS, LPC, MG, and FA, among others. Differentially expressed lipid metabolisms were identified using the DESeq R package functions estimateSizeFactors and nbinomTest. A *p*‐value of < 0.05 and fold changes of > 2 or < 0.5 were set as the threshold values for significant differential expression. Enrichment analysis of differentially expressed lipid metabolic pathways was conducted using R language based on the hypergeometric distribution model, and two‐tailed Student's *t*‐test was further employed to verify the significance of inter‐group metabolite differences.

### 2.13 Molecular Docking Process

Using the RCSB Protein Data Bank database, the crystallographic structure of the PPAR‐α ligand‐binding domain (LBD, PDB accession code: 2ZNN) was obtained. Prior to docking analysis, the protein structure underwent preprocessing which included elimination of solvent molecules and non‐protein components, followed by incorporation of polar hydrogens and application of Kollman atomic partial charges utilizing AutoDock Tools software. The three‐dimensional conformation of SALB was acquired from PubChem database (CID:11629084), with subsequent assignment of Gasteiger partial charges and identification of rotatable bonds. Docking calculations were done using AutoDock Vina (version 1.2.1) with an exhaustiveness parameter of 32 to ensure complete sampling of conformational space. A cubic grid box measuring 60 × 60 × 60 Å was positioned to encompass the established ligand‐binding pocket of PPAR‐α. From the 10 generated binding poses, the most thermodynamically stable conformation was determined by selecting the pose exhibiting the minimal binding free energy (Δ*G*). Visualization and characterization of molecular interactions were done using PyMOL molecular graphics system (version 3.1).

### 2.14 Surface Plasmon Resonance Affinity Characterization Assay

Recombinant human PPAR‐α LBD protein was immobilized on the surface of a Series S Sensor Chip CM5 (Cytiva Sweden; Cat. No. 29149603), with a final immobilization level of approximately 5000 response units. The assay was performed using running buffer. SALB was serially diluted in running buffer to generate the following concentration gradient: 0.19, 0.39, 0.78, 1.56, 3.13, 6.25, 12.5, 25, 50, 100, and 200 μM. All samples were sequentially flowed through the sample channel and reference channel of the chip at a flow rate of 30 μL/min, with a contact time of 90 s and a dissociation time of 120 s. Experimental data were analyzed using Biacore Insight Evaluation Software, and fitting analyses were performed using the 1:1 binding kinetic model and steady‐state affinity model, respectively.

### 2.15 Statistical Analysis

Each experiment was performed in triplicate, and the data are expressed as mean ± standard deviation. The statistical analyses were performed using GraphPad Prism (version 9.5.0; GraphPad, San Diego, CA, USA). Intergroup differences were analyzed using Student's *t*‐test. One‐way analysis of variance with Tukey's post hoc test was used for multiple‐group comparisons. Statistical significance was set at values of *p* < 0.05.

## 3 Results

### 3.1 Network Pharmacology Analysis

**FIGURE 1:**
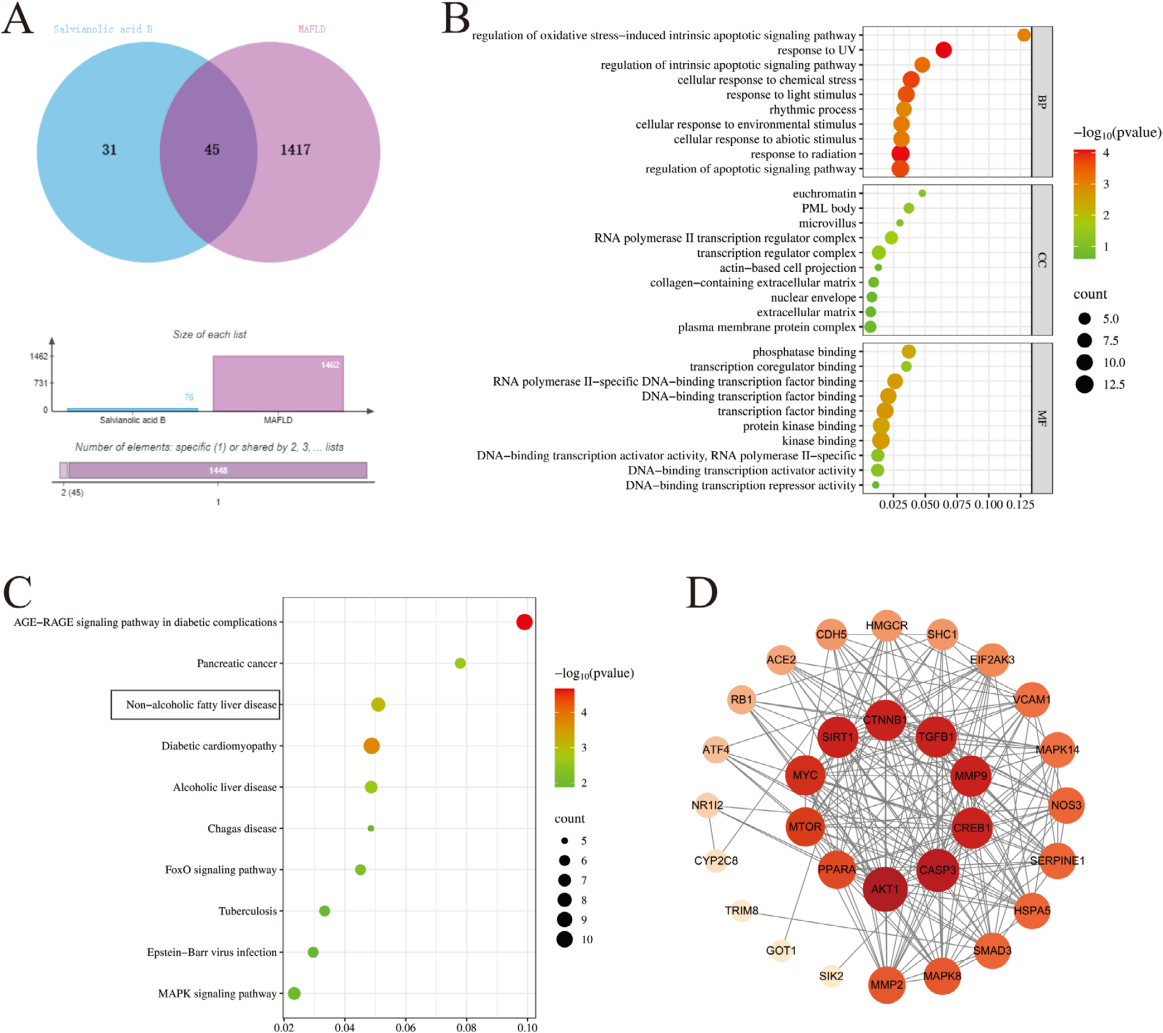
Network pharmacology analysis. (A) Venn diagram of the targets in SALB and MAFLD. (B) Gene ontology enrichment analysis. (C) Top 10 Kyoto Encyclopedia of Genes and Genomes enrichment analysis. (D) Core targets of protein–protein interaction network.

The targets of SALB and MAFLD were obtained from TCMSP and GeneCards, and the corresponding target counts are shown in Table S1. Following normalization using UniProt (http://www.uniprot.org/), 76 SALB‐related targets were identified. The related disease targets were obtained from the GeneCards database, which yielded 1462 targets after excluding duplicates. Finally, 45 STR targets associated with therapeutic effects on liver fibrosis were identified using Venny 2.1 (Figure 1A). Based on these 45 overlapping targets, we determined the relationship between SALB and MAFLD treatment targets. To visualize this relationship, we enriched KEGG and GO analyses using Metascape, obtaining 117 KEGG pathways, 614 GO biological processes (BPs), 14 GO cellular components (CCs), and 31 GO molecular functions (MFs). Figure 1B shows the results of the top 10 KEGG pathway enrichment analyses, which mainly included non‐alcoholic fatty liver disease, the AGE‐RAGE signaling pathway in diabetic complications, and diabetic cardiomyopathy, revealing that SALB may have important therapeutic potential for glycolipid metabolism‐related diseases such as MAFLD. After GO enrichment analysis was performed on these targets, the top 10 GO terms (Figure 1C) were identified (BP, CC, and MF), including regulation of apoptotic signaling pathway (GO:2001233), regulation of intrinsic apoptotic signaling pathway (GO:2001242), and regulation of oxidative stress‐induced intrinsic apoptotic signaling pathway (GO:1902175). A visualized PPI network was established using the STRING database and Cytoscape to identify key targets. The key targets were ranked according to their degree values, and the top ten core targets were identified as AKT1, CASP3, CREB1, MMP9, TGFB1, CTNNB1, SIRT1, MYC, MTOR, and PPARA (Figure 1D). Disturbed lipid metabolism is an important mechanism underlying the pathogenesis of MAFLD. Based on a literature survey, we found that previous studies on SALB in MAFLD have focused on ameliorating inflammation and apoptosis, whereas its effect on lipid metabolism has not been studied. Combined with the results of network pharmacology studies, we propose a reasonable hypothesis that SALB may improve lipid metabolism via PPAR‐α in the treatment of MAFLD.

### 3.2 SALB Alleviated OA and PA‐Induced Lipid Deposition in HepG2 Cells

**FIGURE 2:**
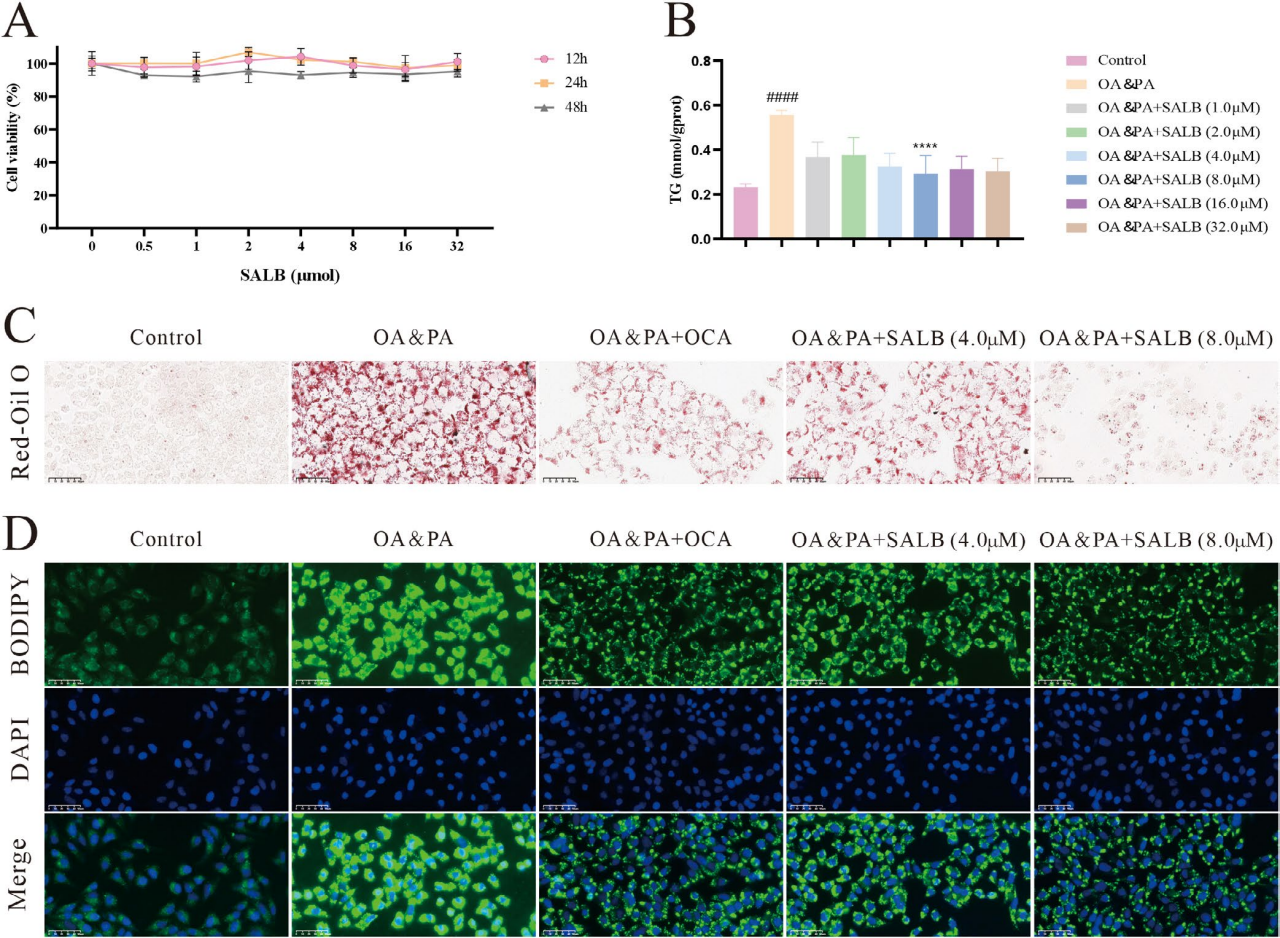
SALB alleviated OA and PA‐induced lipid deposition in HepG2 cells. (A) The cells were treated with SALB (0–32 μM) for 12, 24 or 36 h, and then the cell viability was determined by CCK‐8 assay (*n* = 6). (B) TG levels (*n* = 4). (C) Representative images of Red‐Oil O (400×). (D) Representative images of BODIPY (400×). The data are shown as mean ± standard error of the mean. ^####^ *p* < 0.0001 vs. control group; ****p < 0.0001 vs. OA&PA group.

First, we assessed the effect of SALB on cell viability using the CCK‐8 assay. As shown in Figure 2A, the viability of HepG2 cells was not significantly affected by SALB concentrations ranging from 0.5 to 32 μM after 12, 24, and 48 h of intervention, suggesting that SALB has a good safety profile. To confirm the appeal hypothesis, HepG2 cells were stimulated with OA and PA (Figure 2B). SALB effectively improved TG levels in HepG2 cells, and the effect was best at a concentration of 8 μM. Subsequently, we verified and compared the effects of SALB on lipid deposition in HepG2 cells using Oil Red O and BODIPY staining (Figure 2C,D) and found that SALB significantly improved lipid deposition in HepG2 cells.

### 3.3 SALB Enhanced OA and PA‐Induced Mitochondrial FAO in HepG2 Cells by Promoting PPAR‐α Activation

**FIGURE 3:**
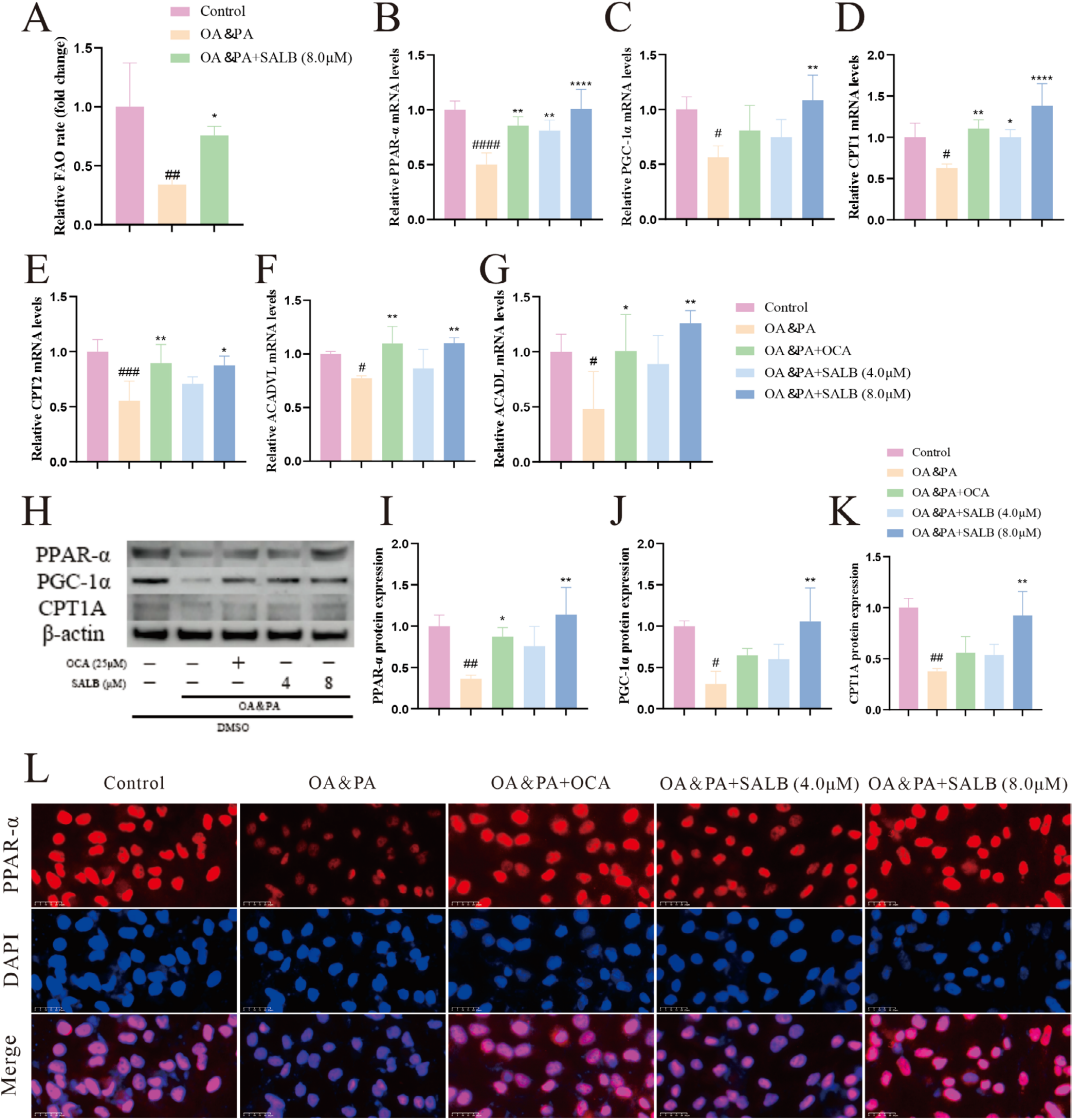
SALB enhanced OA and PA‐induced mitochondrial FAO in HepG2 cells by promoting PPAR‐α activation. (A) Relative fatty acid oxidation rate (*n* = 4). (B–G) Effects of SALB on FAO response‐related mRNA levels by qPCR in HepG2 cells (*n* = 4). (H–K) Effects of SALB on PPAR‐α, PGC‐1α, and CPT1A protein expressions by western blotting in each group (*n* = 3). (L) Representative images of IF staining (800×). The data are shown as mean ± standard error of the mean. ^#^ *p* < 0.05, ^##^ *p* < 0.01, ^###^ *p* < 0.001, ^####^ *p* < 0.0001 vs. control group; *p < 0.05, **p < 0.01, ****p < 0.0001 vs. OA&PA group.

To validate the effect of SALB on MAFLD lipid metabolism, we performed an FAO assay and found that SALB significantly restored the FAO levels in HepG2 cells (Figure 3A). The nuclear receptor PPAR‐α is a key target involved in the regulation of mitochondrial FAO and is essential for lipid metabolism homeostasis, which we further verified using qPCR, western blotting, and immunofluorescence. The relative mRNA levels of PPAR‐α were downregulated after incubation with OA and PA, whereas SALB treatment restored their expression, particularly at a concentration of 8 μM (Figure 3B). Similarly, FAO response‐related mRNA levels of *PGC‐1α, CPT1, CPT2, Acadvl*, and *Acadl* were suppressed by OA and PA and upregulated by SALB (Figure 3C–G). Western blotting showed that PPAR‐α, PGC‐1α, and CPT1A protein expression levels were strongly downregulated by OA and PA and upregulated by SALB (Figure 3H–K). Immunofluorescence also demonstrated that PPAR‐α was downregulated by OA and PA and upregulated by SALB, consistent with the western blotting results (Figure 3L). These results confirmed that SALB activates PPAR‐α and mediates FAO in vitro.

### 3.4 SALB Reduced Lipid Deposition in HFD Mice

**FIGURE 4:**
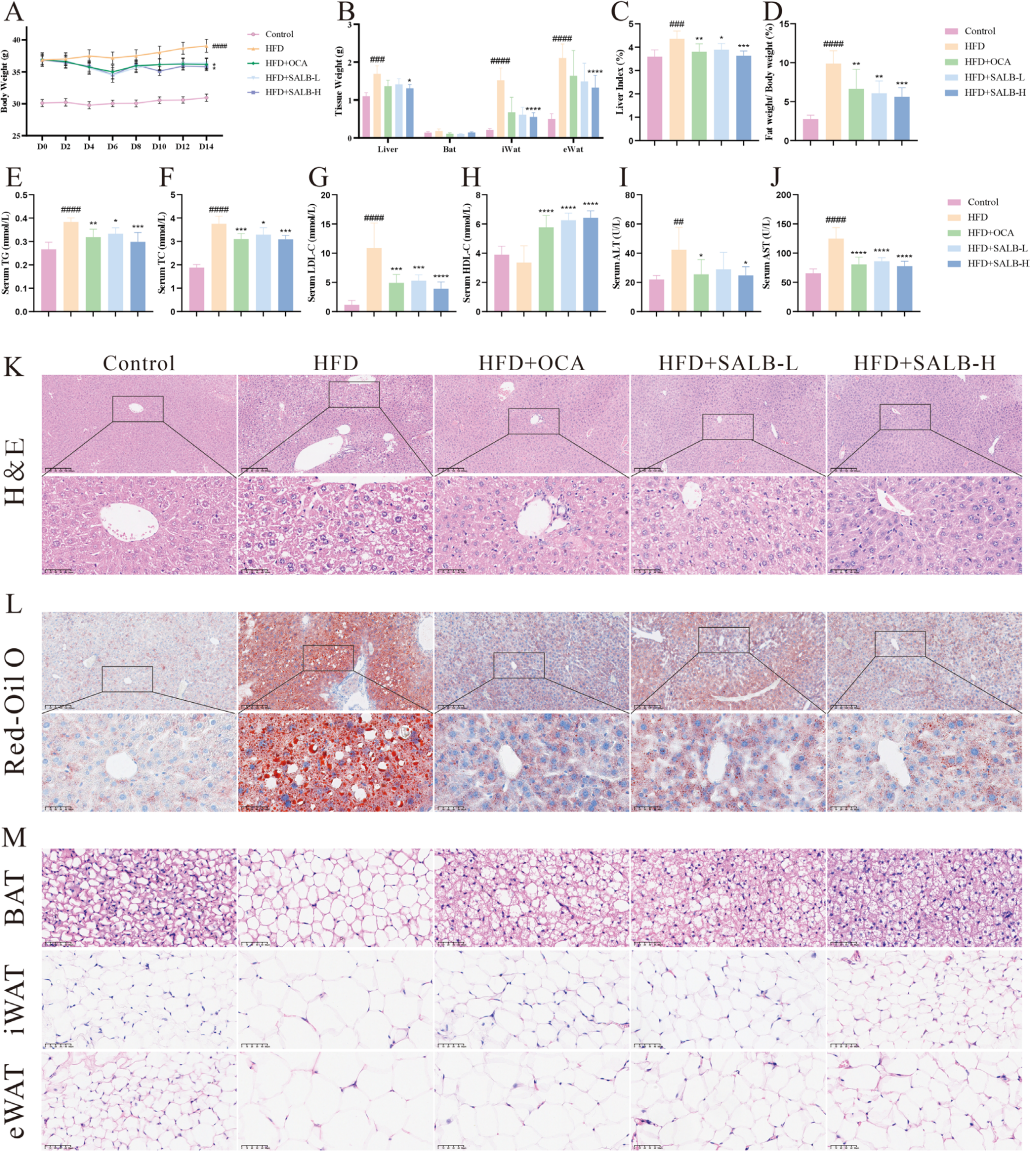
SALB reduced lipid deposition in HFD mice. (A) Body weight changes (*n* = 6). (B–D) Tissue weight, liver index, and fat index (*n* = 6). (E–J) Serum TG, TC, LDL‐C, HDL‐C, ALT, and AST levels (*n* = 6). (K, L) Representative images of hematoxylin and eosin and oil red O of livers in each group (100×, 400×). (M) Representative images of hematoxylin and eosin of BAT, IWAT, and EWAT in each group (400×). The data are shown as mean ± standard error of the mean. ^*#*#^ *p* < 0.01, ^*##*#^ *p* < 0.001, ^####^ *p* < 0.0001 vs. control group; *p < 0.05, **p < 0.01, ***p < 0.001, ****p < 0.0001 vs. HFD group.

SALB treatment markedly reduced HFD‐induced weight gain (Figure 4A). Furthermore, tissue weights, including those of the liver and inguinal and epididymal white adipose tissues, were markedly reduced by SALB (Figure 4B), similar to the liver and fat indices (Figure 4C,D). The administration of SALB resulted in a reduction in serum TG and TC levels in mice, as well as a decrease in serum LDL‐C levels (Figure 4E–G). In contrast, the serum HDL‐C levels increased after SALB administration (Figure 4H). In addition, serum ALT and AST levels were markedly reduced by SALB treatment (Figure 4I,J). Pathological changes in the liver tissue also improved following SALB treatment, especially in the HFD + SALB‐H group, as shown by H&E and Oil Red O staining. Significantly fewer liver lipid vacuoles and lipid droplets were observed compared to the vehicle‐treated HFD group (Figure 4K,L). Furthermore, pathological analysis indicated that BAT subjected to intervention exhibited a multicompartmental morphology, stronger red coloration, and higher prevalence of small lipid droplets, whereas HFD mice showed a mononuclear morphology and accumulation of large lipid droplets (Figure 4M). In addition, HFD mice presented with a substantial number of larger adipocytes in both inguinal and epididymal white adipose tissue cells, whereas SALB treatment significantly reduced adipocyte size. These results indicate that SALB reduced lipid deposition in vivo.

### 3.5 SALB Ameliorates HFD‐Induced Hepatic Lipid Metabolic Dysregulation via PPAR‐α Modulation

**FIGURE 5:**
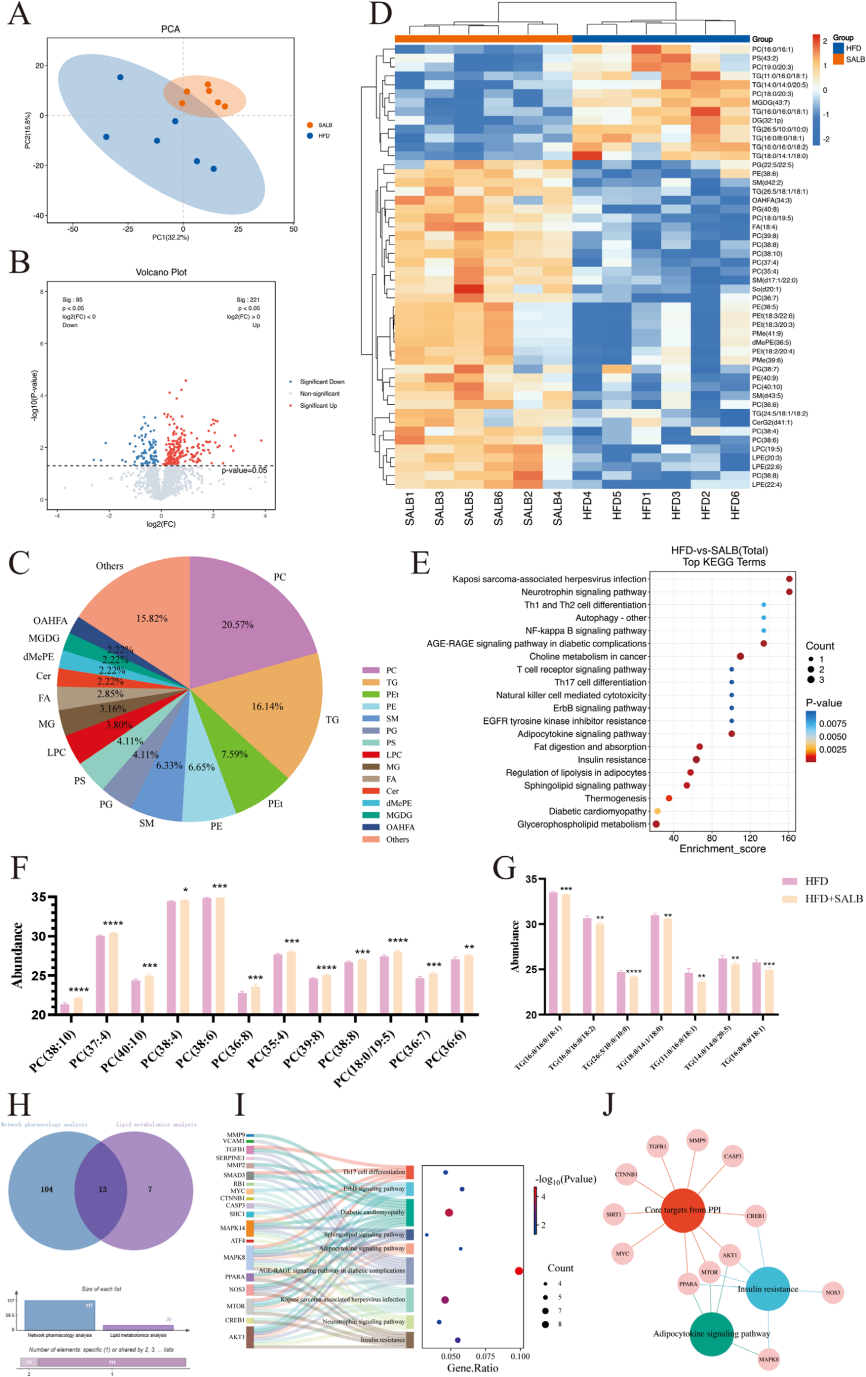
SALB ameliorates HFD‐induced hepatic lipid metabolic dysregulation via PPAR‐α modulation. (A) Principal component analysis (PCA) plots of lipid metabolisms. (B) Volcano plot of differentially expressed lipid metabolisms. (C) Composition ratio of different lipid metabolites. (D) Heatmaps of the expression of significant lipid class. (E) KEGG pathway analysis. (F, G) Relative abundance of PCs and TGs. (H) Venn diagram of lipidomics and network pharmacology‐enriched KEGG pathways. (I) Overlapping KEGG pathways (*p* < 0.05). (J) Intersection of PPI core targets and lipid metabolism‐related pathway targets. The data are shown as mean ± standard error of the mean. **p < 0.01, ***p < 0.001.

To validate the potential mechanism of action of SALB in lipid metabolism, we performed lipid metabolomics using LC/MS. The lipid composition of liver tissues was significantly different between the HFD and SALB groups (Figure 5A). The multi‐dimensional and single‐dimensional analysis results showed that 316 lipid metabolisms were differentially expressed (variable importance of projection > 1, *q* < 0.05) in the SALB‐treated mice (Figure 5B), with 95 downregulated and 221 upregulated. Concurrently, the results revealed that among these 316 differentially expressed lipid metabolites, the predominant changes were observed in the levels of PCs (20.57%) and TGs (16.14%) (Figure 5C). Analysis of the top 50 lipid metabolites with the largest differences in lipid metabolism (Figure 5D) indicated that these differences were primarily attributable to subtle lipid components. The KEGG pathway analysis results showed that SALB mainly influenced lipid metabolism‐ and inflammation‐related pathways, including fat digestion and absorption, regulation of lipolysis in adipocytes, thermogenesis, and the NF‐κB signaling pathway (Figure 5E). Furthermore, we focused on PCs and TGs as the predominantly altered differential lipid metabolites among the top 50 hits. Previous studies have demonstrated that PCs possess antioxidant and anti‐inflammatory potential and serve as key components of very low‐density lipoproteins (VLDLs)—the primary vehicles for transporting triglycerides from the liver to extrahepatic tissues. As illustrated in Figure 5F,G, SALB significantly increased hepatic PC levels while markedly decreasing TG content in liver tissue. These results suggest that SALB plays a key role in MAFLD treatment via lipid metabolism‐ and inflammation‐related pathways.

To further decipher the potential mechanism by which SALB alters lipid metabolism, a combined analysis of lipid metabolomics and network pharmacology results was conducted. By intersecting the potential KEGG pathways identified from lipid metabolomics with those from network pharmacology, 13 common pathways were obtained (Figure 5H), and pathways with *p* < 0.05 are presented in Figure 5I. Considering that the insulin resistance and adipocytokine signaling pathways are closely associated with lipid metabolism, these two pathways were integrated with PPI results for subsequent analysis. This integrated approach identified PPAR‐α, MTOR, AKT1, and CREB1 as the four core targets mediating SALB's regulation of lipid metabolism (Figure 5J).

### 3.6 SALB Enhanced Lipid Metabolism Through the PPAR‐α/PGC‐1α Pathway in HFD Mice

**FIGURE 6:**
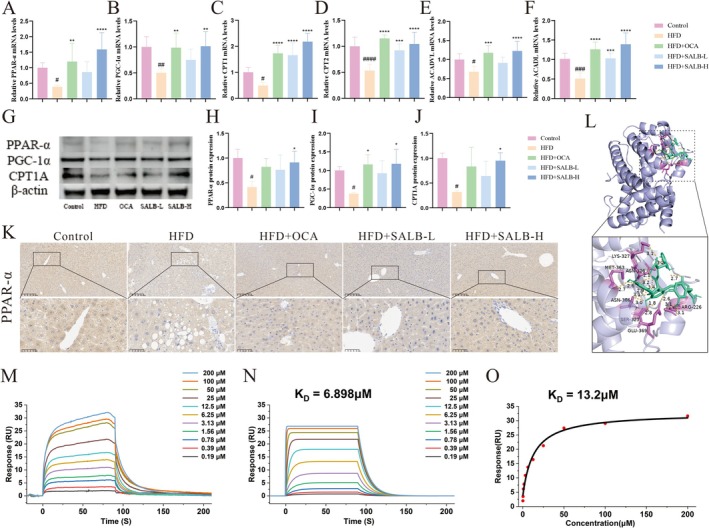
SALB enhanced lipid metabolism through the PPAR‐α/PGC‐1α pathway in HFD mice. (A–F) Effects of SALB on FAO response‐related mRNA levels by qPCR in each group (*n* = 6). (G–J) Effects of SALB on PPAR‐α, PGC‐1α, and CPT1A protein expressions by western blotting in each group (*n* = 3). (K) Representative images of immunohistochemical hepatic PPAR‐α staining in each group (400×). (L) Molecular docking conformation of SALB with PPAR‐α. (M) The binding affinities of recombinant SALB to PPAR‐α were measured by SPR assay. (N) SPR results of kinetic fitting. (O) SPR results of steady‐state fitting. The data are shown as mean ± standard error of the mean. ^#^ *p* < 0.05, ^*#*#^ *p* < 0.01, ^*##*#^ *p* < 0.001, ^####^ *p* < 0.0001 vs. control group; *p < 0.05, **p> < 0.01, ***p < 0.001, ****p < 0.0001 vs. HFD group.

The SALB‐treated groups showed enhanced expression of FAO‐related genes compared to the HFD group (Figure 6A–F). Western blotting showed that the HFD significantly reduced PPAR‐α, PGC‐1α, and CPT1A expression in the liver, whereas treatment with SALB restored these levels (Figure 3G–J). Furthermore, IHC staining demonstrated that PPAR‐α expression in the HFD group was alleviated in the SALB treatment group, consistent with the western blotting results (Figure 6K). These results suggest that SALB enhances PPAR‐α/PGC‐1α signaling pathway activation. To further confirm whether PPAR‐α is a potential target for SALB in the treatment of MAFLD, molecular docking was used to predict the protein‐binding sites between SALB and PPAR‐α (Figure 6L). The binding energy was calculated to be −7.0 kcal/mol, indicating favorable binding. SPR analysis revealed that the binding signal between SALB and PPAR‐α protein exhibited characteristics of “fast association‐slow dissociation” (Figure 6M). Global fitting of the data using a 1:1 binding kinetic model yielded an equilibrium dissociation constant (*K*_D_) of 6.898 μM (Figure 6N). To validate the reliability of the kinetic results, a steady‐state affinity model was simultaneously employed, yielding an apparent *K*_D_ of 13.2 μM (Figure 6O). Notably, persistent residual signal was observed during the dissociation phase, which may have compromised the accurate determination of steady‐state equilibrium levels. Therefore, the kinetic fitting results based on the complete association and dissociation time courses were considered more reliable. These results indicate that SALB may protect against MAFLD through its interaction with PPAR‐α.

### 3.7 SALB Mitigates Oxidative Stress and Inflammation Induced by Excessive Lipid Deposition

**FIGURE 7:**
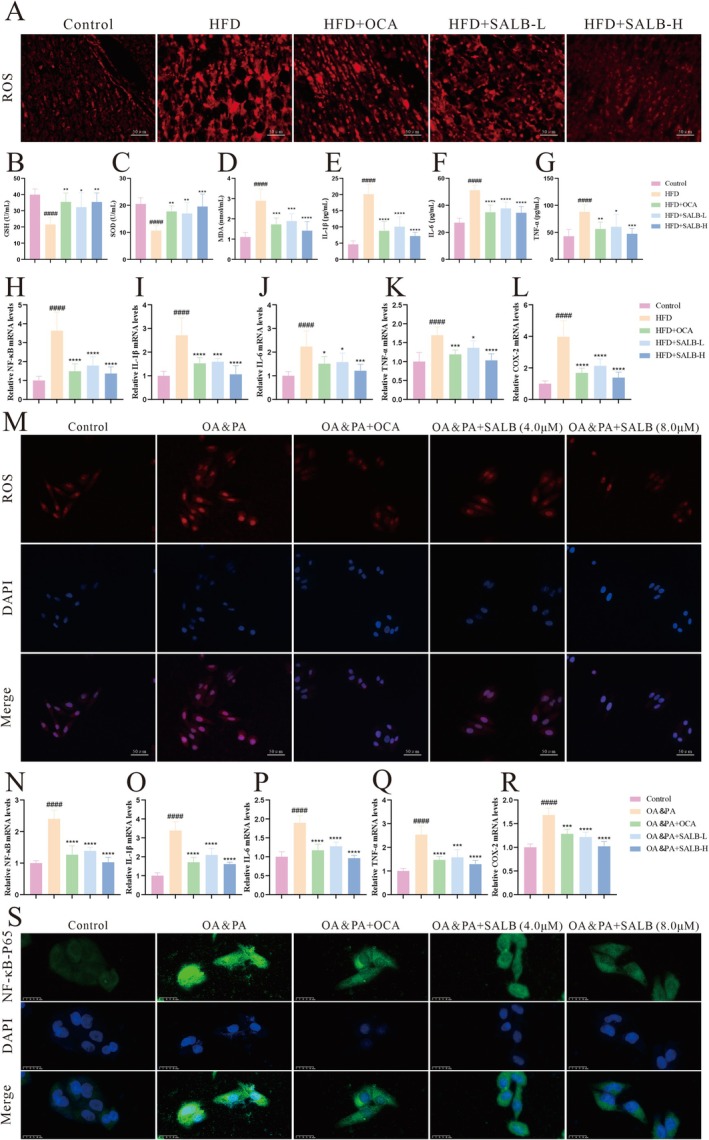
SALB inhibits oxidative stress and inflammation induced by excessive lipid deposition. (A) ROS levels of hepatic tissue (400×). (B–G) Serum GSH, SOD, MDA, IL‐1β, IL‐6, and TNF‐α levels (*n* = 6). (H–L) Effects of SALB on inflammation‐related mRNA levels by qPCR in each group (*n* = 6). (M) ROS levels of HepG2 cells (400×). (N–R) Effects of SALB on inflammation‐related mRNA levels by qPCR in each group of HepG2 cells (*n* = 4). (S) Immunofluorescence staining for NF‐κB P65 expression in HepG2 (800×). The data are shown as mean ± standard error of the mean. ^####^ *p* < 0.0001 vs. control group; *p < 0.05, **p < 0.01, ***p < 0.001, ****p < 0.0001 vs. HFD group.

According to the “multiple hit” hypothesis of MAFLD, the second hit encompasses lipid peroxidation and oxidative stress within hepatocytes burdened by excessive lipid accumulation, culminating in mitochondrial dysfunction and the production of proinflammatory cytokines. Lipidomic findings reveal the potential of SALB to suppress oxidative stress and the NF‐κB inflammatory signaling pathway, though these effects requires further validation. Compared with the HFD group, the SALB‐treated group exhibited significantly reduced hepatic ROS levels, along with markedly increased serum GSH and SOD levels, and decreased serum MDA levels (Figure 7A–D). Consistent with our expectations, ELISA results demonstrated that serum levels of the proinflammatory cytokines IL‐1β, IL‐6, and TNF‐α were significantly lower in the SALB‐treated group compared with the HFD group (Figure 7E–G). Furthermore, SALB significantly downregulated the mRNA expression levels of NF‐κB, IL‐1β, IL‐6, TNF‐α, and COX‐2 in liver tissue (Figure 7H–L). These findings were corroborated by in vitro experiments using OA & PA‐induced HepG2 cells (Figure 7M–R). Concurrently, IF staining revealed that P65 was highly expressed with prominent nuclear translocation in OA&PA‐induced HepG2 cells, while SALB effectively reverse this phenomenon (Figure 7S). Collectively, these results suggest that SALB mitigates oxidative stress induced by excessive lipid deposition and suppresses the NF‐κB inflammatory signaling pathway, thereby exerting a multi‐faceted protective effect against MAFLD.

### 3.8 PPAR‐α Antagonist Significantly Eliminated the SALB‐Mediated Enhancements in Lipid Metabolism and Suppression of Inflammation in HepG2 Cells

**FIGURE 8:**
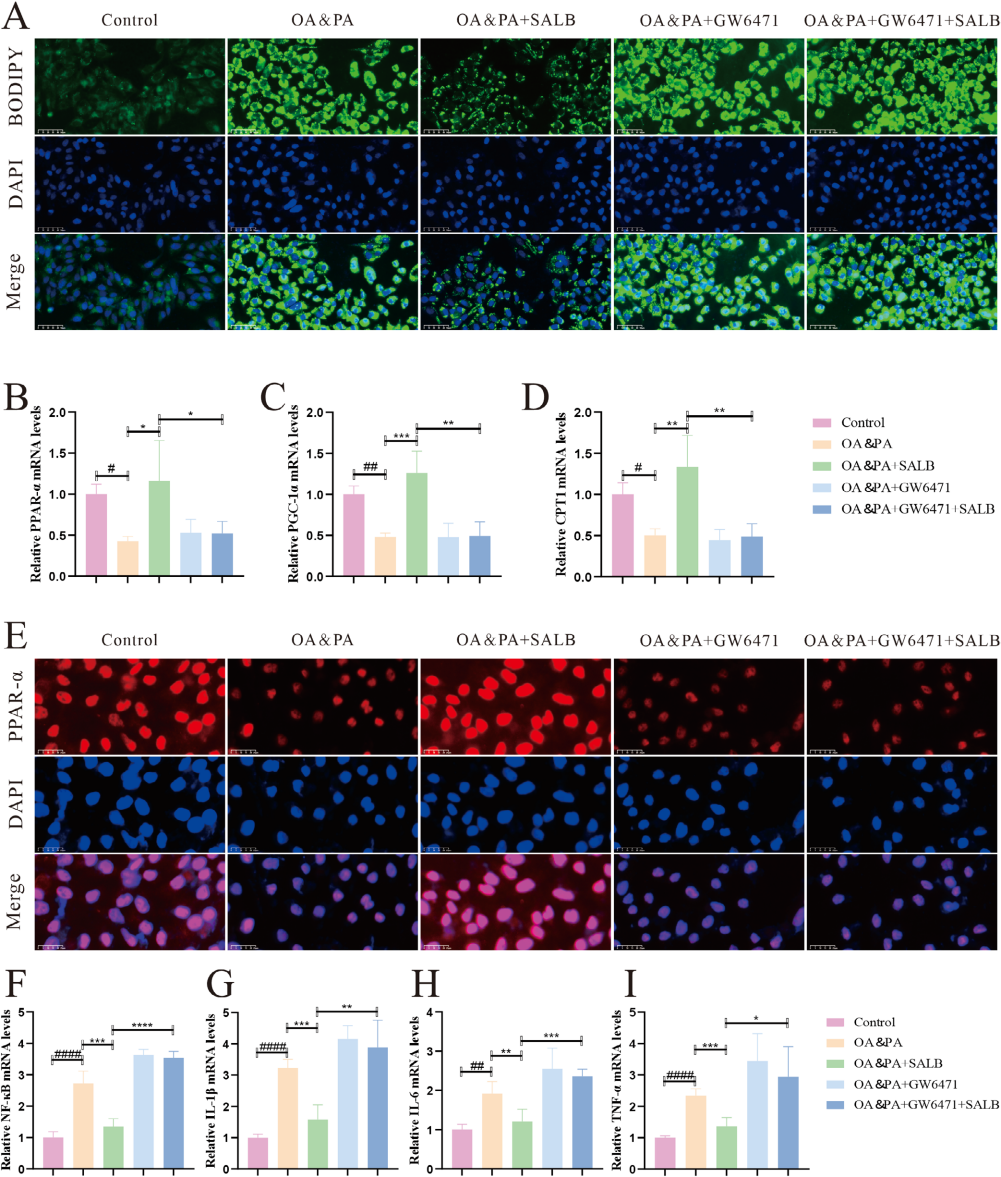
PPAR‐α antagonist partially inhibited the lipid metabolism‐enhancing effect of SALB on HepG2 cells. (A) Representative images of BODIPY (400×). (B–D) Effects of SALB on FAO response‐related mRNA levels by qPCR in each group (*n* = 4). (E) Immunofluorescence staining for PPAR‐α expression in HepG2 (800×). (F–I) Inflammation‐related mRNA levels by qPCR in each group (*n* = 4). The data are shown as mean ± standard error of the mean. ^*#*^ *p* < 0.05, ^##^ *p* < 0.01 vs. control group; *p < 0.05, **p < 0.01, ***p < 0.001.

To explore the role of PPAR‐α in the lipid‐lowering effect of SALB, HepG2 cells were treated with SALB with or without the PPARα inhibitor GW6471. The BODIPY results showed that GW6471 exacerbated lipid accumulation following SALB treatment (Figure 8A). qPCR and immunofluorescence results showed that GW6471 notably decreased the protective effect of SALB against OA‐ and PA‐induced suppression of PPAR‐α, PGC‐1α, and CPT1 expression (Figure 8B–E). Furthermore, GW6471 significantly abrogated the SALB‐mediated suppression of the NF‐κB inflammatory signaling pathway (Figure 8F–I). Collectively, these findings indicate that SALB targets PPAR‐α to ameliorate lipid metabolism, improve mitochondrial function, and inhibit oxidative stress and inflammatory responses.

## 4 Discussion

Dysregulated lipid metabolism is a pivotal contributor to MAFLD (Badmus et al. 2022), and nuclear receptor PPAR‐α‐mediated mitochondrial fatty acid β‐oxidation has emerged as a promising therapeutic target for MAFLD management. Emerging evidence supports a bidirectional regulatory role of PPAR‐α in MAFLD progression. On one hand, PPAR‐α enhances fatty acid oxidation by activating downstream target genes including CPT1 and ACADL, thereby attenuating intrahepatic TG accumulation (Ni et al. 2022). On the other hand, PPAR‐α suppresses NF‐κB signaling pathway activation to mitigate hepatic inflammation (Gervois and Mansouri 2012; Lyu et al. 2025; Niu et al. 2024; Wang et al. 2025; Xiong et al. 2024). Notably, the vicious cycle between inflammation and lipid metabolic dysregulation serves as a key driver of MAFLD progression (Heeren and Scheja 2021).

Although accumulating reports have highlighted the beneficial effects of SALB on inflammation, oxidative stress, and gut microbiota modulation, its specific role in MAFLD treatment remains incompletely understood. To address this gap, we first employed network pharmacology to identify potential therapeutic targets. By matching drug‐ and disease‐related targets, we screened 45 candidate targets, with KEGG and GO enrichment analyses highlighting associations with MAFLD, inflammation, and oxidative stress. PPI analysis further identified PPAR‐α as a core target, leading to our central hypothesis: SALB restores lipid metabolic homeostasis through PPAR‐α‐mediated FAO, thereby exerting a therapeutic reversal effect on MAFLD.

Multiple lines of evidence from in vitro and in vivo experiments supported this hypothesis. In OA&PA‐induced HepG2 cells, SALB reversed steatosis, diminished intracellular lipid droplet accumulation, and inhibited TG elevation, which validating its therapeutic reversal properties. The HepG2 cell line was selected for its ease of culture, stable genetic background, and similarity to healthy human hepatocytes (Gerets et al. 2012). However, limitations inherent to this hepatocellular carcinoma‐derived model (e.g., reduced differentiation, altered expression of liver‐specific metabolic enzymes (Gerets et al. 2012), and inherent signaling pathway abnormalities) should be acknowledged. Thus, mechanistic findings in HepG2 cells require further validation in primary hepatocytes or alternative models to reflect physiological conditions. In HFD‐induced MAFLD mice, SALB intervention significantly improved body weight and obesity index abnormalities, downregulated serum TC, TG, and LDL‐C levels, and reduced hepatic steatosis severity as confirmed by histopathology. These findings demonstrated SALB's ability to reverse established MAFLD pathological phenotypes.

Mechanistically, OA&PA‐induced HepG2 cell experiments revealed that SALB upregulated PPAR‐α, the key regulator of mitochondrial biogenesis and function PGC‐1α, and FAO‐related enzymes (CPT1 and ACADL). These results indicate that SALB enhances lipid metabolism and mitochondrial function by activating the PPAR‐α/PGC‐1α pathway. Consistent in vivo findings showed SALB‐induced upregulation of hepatic PPAR‐α and its downstream target genes in HFD‐fed mice, confirming that SALB can effectively activate the PPAR‐α/PGC‐1α signaling pathway, which may represent the potential mechanism underlying SALB's improvement of MAFLD.

Lipid metabolomics analysis further complemented these mechanistic insights, revealing significant alterations in hepatic lipid metabolites following SALB treatment, with the most prominent changes observed in PCs (20.57%) and TGs (16.14%). KEGG pathway enrichment analysis linked these metabolites to lipid metabolism and inflammation‐related pathways, including fat digestion and absorption, regulation of adipocytes, thermogenesis, and the NF‐κB signaling pathway. Notably, PCs serve as key components of VLDLs, which are the primary carriers for hepatic TG export (Kamanna and Kashyap 2000; Takahashi et al. 2003), and their synthesis impairment in MAFLD inhibits VLDL secretion, exacerbating intrahepatic lipid accumulation, inflammation, and fibrosis (Jiang et al. 2022; Song et al. 2025). SALB‐induced PC upregulation may therefore facilitate VLDL synthesis and secretion, accelerating TG export and alleviating steatosis; these may synergize with PPAR‐α‐mediated FAO promotion to collectively ameliorate lipid metabolic disorders. Additionally, PC's anti‐inflammatory and antioxidant properties (Melo et al. 2018; Yang et al. 2020) suggest a supplementary mechanism by which SALB modulates oxidative stress and inflammation in MAFLD.

In MAFLD, hepatic lipid accumulation precedes hepatitis, lipid peroxidation, and apoptosis (Tong et al. 2023), with oxidative stress primarily triggered by excessive free fatty acids (Clare et al. 2022; Cui et al. 2025). FAO represents the primary pathway for fatty acid degradation (Houten et al. 2016; Peng et al. 2024). Impairment of mitochondrial FAO not only leads to intracellular lipid accumulation in hepatocytes but also induces ROS release and oxidative damage (Li, Gao, et al. 2025). This scenario further activates NF‐κB, promoting the gene expression of proinflammatory cytokines such as IL‐1β, IL‐6, TNF‐α, and COX‐2 (Delerive et al. 1999), thereby exacerbating the progression of MAFLD. Integrated network pharmacology and lipid metabolomics analysis identified 13 shared KEGG pathways and four core targets (PPAR‐α, MTOR, AKT1, CREB1) underlying SALB's lipid metabolic regulation. While in vitro and in vivo experiments confirmed SALB‐induced PPAR‐α upregulation, direct evidence for PPAR‐α activation remained limited.

Molecular docking analysis and SPR represented critical approaches to validate the direct interaction between SALB and PPAR‐α. Molecular docking predicted favorable binding affinity between SALB and PPAR‐α. Further analysis of key amino acid residues revealed that the binding of SALB to PPAR‐α was primarily mediated through the formation of an extensive interaction network with critical residues in the PPAR‐α LBD, including LYS‐327, ASN‐326, MET‐363, and GLU‐369, thereby stabilizing its binding in the active pocket. Specifically, binding distance data indicated that dense hydrogen bond interactions (2.5–3.5 Å) were formed between SALB and these residues, which provided crucial recognition specificity and binding strength for the interaction. These intermolecular interactions indicated that SALB could effectively bind to PPAR‐α and induce its activation, which was highly consistent with the biological observations. SPR experiments further confirmed specific binding between SALB and PPAR‐α, with a kinetic fitting‐derived KD of 6.898 μM, which is classified as strong binding in protein‐small molecule interactions. Steady‐state fitting yielded an apparent KD of 13.2 μM, but persistent residual signals during dissociation compromised its reliability, rendering kinetic fitting results more robust. Collectively, these findings provided direct molecular‐level evidence for the hypothesis that PPAR‐α serves as a key target of SALB.

Previous studies have demonstrated that PPAR‐α reduces proinflammatory cytokine release and mitigates ROS production by inhibiting the NF‐κB signaling pathway (Reda et al. 2023; Xiong et al. 2024), both of which are core components of the “second hit” in MAFLD progression. Further investigations in the present study revealed that SALB significantly suppressed ROS levels and inflammatory responses, while concurrently downregulating NF‐κB signaling pathway expression, in both in vitro and in vivo models. This effect is likely mediated through the combined actions of PPAR‐α‐dependent direct inhibition (Reda et al. 2023; Xiong et al. 2024) and PC‐mediated anti‐inflammatory activity.

To confirm PPAR‐α dependency, we used the PPAR‐α inhibitor GW6471 to examine the protective effect of SALB on lipid deposition and its effect on the PPAR‐α/PGC‐1α and NF‐κB signaling pathway. As expected, we found that GW6471 significantly abrogated the SALB‐mediated improvements in lipid metabolism. Furthermore, GW6471 markedly reversed the SALB‐induced upregulation of PPAR‐α downstream target genes such as PGC‐1α and CPT1, as well as the suppression of the NF‐κB signaling pathway. These results demonstrate, for the first time, that SALB targeting PPAR‐α protects against MAFLD invasion.

Notably, this study carries several limitations that warrant acknowledgment. First, the present study did not include a direct comparative analysis between SALB and other natural or synthetic PPAR‐α agonists. Additionally, hepatocellular uptake kinetics, tissue distribution, pharmacokinetics, potential off‐target effects, and combined metabolite analysis of SALB remain unexplored. Mechanistically, the direct interaction between SALB and PPAR‐α requires further investigation using genetic intervention approaches such as PPAR‐α knockout mouse models. Meanwhile, the functional role of SALB in adipocyte metabolism, as uncovered by integrated network pharmacology and lipid metabolomics analyses, warrants further exploration.

These unresolved questions represent a critical focus for future research to fully elucidate SALB's underlying mechanisms and translational potential. In contrast to other plant‐derived PPAR‐α agonists, this study is the first to confirm direct SALB‐PPAR‐α binding via SPR and identify SALB's unique role in bridging PPAR‐α activation with VLDL secretion through PC and TG modulation. The dual mechanism of PPAR‐α‐mediated NF‐κB inhibition and lipid metabolism‐driven anti‐inflammation further distinguishes SALB as a promising candidate for MAFLD therapy. Collectively, our findings identify PPAR‐α as the key functional target of SALB in MAFLD, providing a clear target‐oriented mechanism for its anti‐MAFLD efficacy.

## 5 Conclusion

**FIGURE 9:**
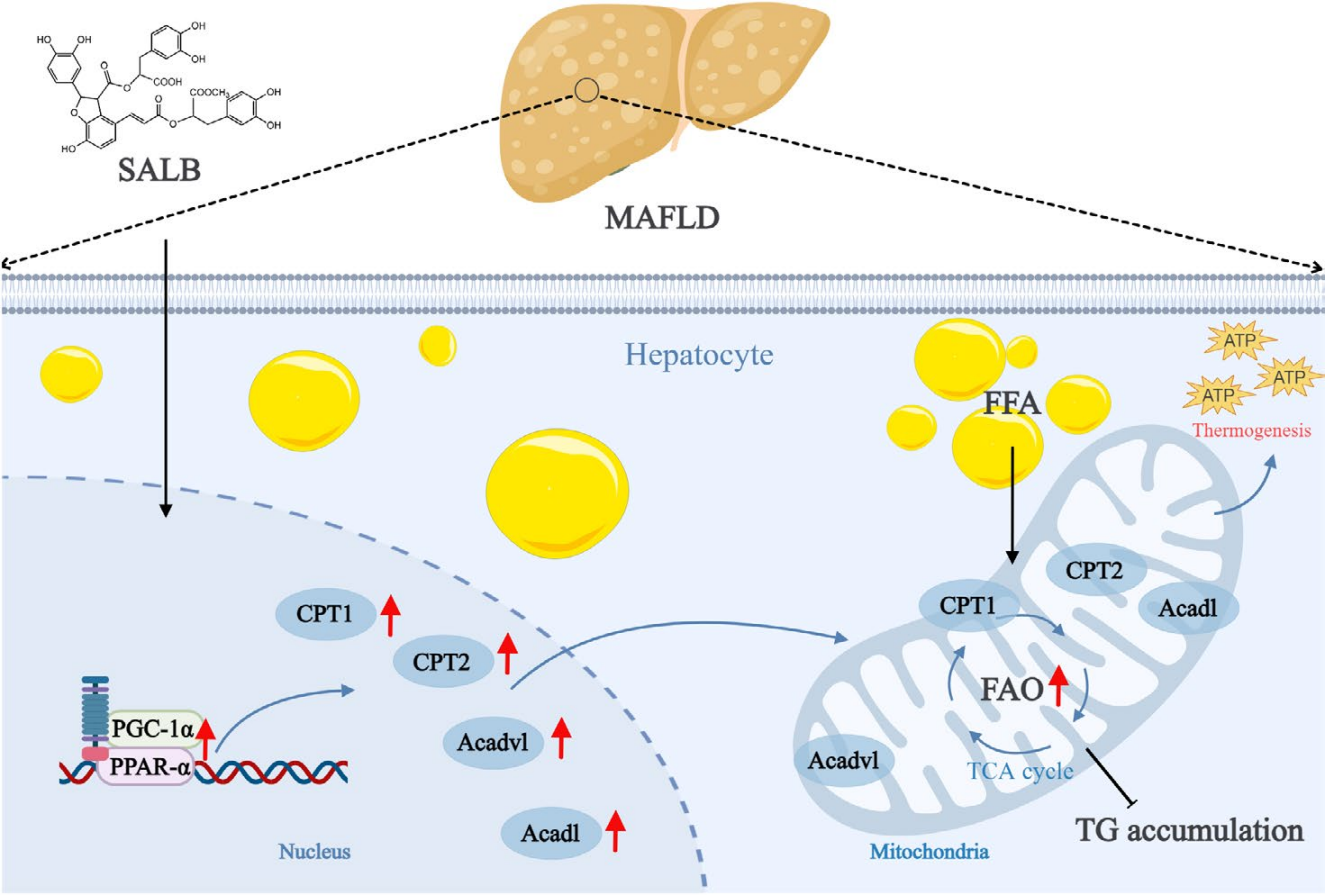
The potential mechanism of SALB on MAFLD.

In this study, integrating network pharmacology and lipidomics, we elucidated the molecular mechanisms underlying the ameliorative effects of SALB, a major bioactive component of a traditional Asian health‐promoting food. Supported by molecular docking, SPR assays, and PPAR‐α inhibitor validation, our findings demonstrate that SALB acts as a novel direct PPAR‐α agonist. It exerts therapeutic potential through activating the PPAR‐α/PGC‐1α pathway (Figure 9), modulating PC and TG metabolism, and inhibiting NF‐κB signaling. This multi‐pronged action alleviates lipid metabolic dysregulation, mitigates inflammatory responses, and reverses MAFLD‐related pathological alterations. Notably, this investigation highlights the efficacy of SALB, a natural edible‐medicinal compound, in MAFLD management, while clarifying PPAR‐α as a pivotal regulator mediating its lipid‐lowering and anti‐inflammatory effects via direct binding and pathway activation. These results provide conceptual support for SALB's application in lipid metabolism disorders and establish an integrative research paradigm for exploring bioactive compounds from edible‐medicinal herbs. Further studies are warranted to validate SALB's clinical efficacy and safety, reinforcing its translational potential in MAFLD intervention.

## Author Contributions

**Fengyan Huang:** data curation (equal), formal analysis (equal), writing – original draft (equal). **Chen Qiu:** data curation (equal), formal analysis (equal). **Danna Wang:** data curation (equal), writing – original draft (equal). **Yuanying Ni:** visualization (equal), data curation (equal). **Zhuotao Fu:** visualization (equal). **Linchun Fu:** supervision (equal). **Chao Liang:** conceptualization (equal). **Shangyi Huang:** conceptualization (equal), writing – review editing (equal). **Zhitong Deng:** methodology (equal), supervision (equal), writing – review editing (equal).

## Funding

This work was supported by the Joint Health Science and Technology Innovation Project of Hainan Province (WSJK2025QN098), Hainan Provincial Natural Science Foundation of China (825MS185), the Traditional Chinese Medicine Bureau of Guangdong Province (20261083), Hainan Province Clinical Medical Center and National Construction of Traditional Chinese Medicine Specialty (Massage Department).

## Ethics Statement

All Animal experiments were approved by the Animal Ethics Committee of Guangzhou University of Chinese Medicine (Ethics Certificate number, 20250120005).

## Consent

The authors have nothing to report.

## Conflicts of Interest

The authors declare no conflicts of interest.

## Supporting information


**Table S1:** Count of target genes of SALB.

## Data Availability

The data that support the findings of this study are available from the corresponding author upon reasonable request.
