# Supplementary material for: Salvianolic Acid B Alleviates MAFLD by Targeting PPAR‐α: Mechanistic Insights From Network Pharmacology and Lipidomics
Source: Food Sci Nutr. 2026 Mar 19;14(3):e71646. doi: 10.1002/fsn3.71646 (PMC13093431; doi:10.1002/fsn3.71646)
Supplement: Supplementary file 1 — Table S1: Count of target genes of SALB. [file FSN3-14-e71646-s001.docx]

**Supplement Table 1. Count of target genes of SALB**

| Database | Target |
| --- | --- |
| TCMSP | 0 |
| GeneCards | 76 |
